# Semi-Supervised Instance-Segmentation Model for Feature Transfer Based on Category Attention

**DOI:** 10.3390/s22228794

**Published:** 2022-11-14

**Authors:** Hao Wang, Juncai Liu, Changhai Huang, Xuewen Yang, Dasha Hu, Liangyin Chen, Xiaoqing Xing, Yuming Jiang

**Affiliations:** 1 School of Computer Science, Sichuan University, Chengdu 610065, China; 2 Sichuan GreatWall Computer System Co., Ltd., Luzhou 646000, China; 3 Institute for Industrial Internet Research, Sichuan University, Chengdu 610065, China; 4 College of Aviation Engineering, Civil Aviation Flight University of China, Guanghan 618307, China

**Keywords:** semi-supervised learning, instance segmentation, feature transfer, attention mechanism

## Abstract

In the task of image instance segmentation, semi-supervised instance segmentation algorithms have received constant research attention over recent years. Among these algorithms, algorithms based on transfer learning are better than algorithms based on pseudo-label generation in terms of segmentation performance, but they can not make full use of the relevant characteristics of source tasks. To improve the accuracy of these algorithms, this work proposes a semi-supervised instance segmentation model AFT-Mask (attention-based feature transfer Mask R-CNN) based on category attention. The AFT-Mask model takes the result of object-classification prediction as “attention” to improve the performance of the feature-transfer module. In detail, we designed a migration-optimization module for connecting feature migration and classification prediction to enhance segmentation-prediction accuracy. To verify the validity of the AFT-Mask model, experiments were conducted on two types of datasets. Experimental results show that the AFT-Mask model can achieve effective knowledge transfer and improve the performance of the benchmark model on semi-supervised instance segmentation.

## 1. Introduction

Instance segmentation is a key technology in research fields, such as autonomous driving, biomedical image processing [1], and robot vision control, so it has attracted strong enthusiasm and research attention. Instance-segmentation methods are divided into fully-supervised algorithms, weakly-supervised algorithms and semi-supervised algorithms, which each use different types of image datasets. Among these, fully-supervised algorithms can achieve the best accuracy, but have higher data requirements, so their application and promotion are limited by the higher cost of data collection [2]. The cost of collecting the datasets required by weakly-supervised algorithms is not high, but the accuracy of such algorithms is lower than that of fully-supervised algorithms [3,4]. Semi-supervised algorithms can simultaneously use a complete instance to segment labeled image data, weakly-labeled image data, or unlabeled image data, resulting in better semi-supervised learning performance with a moderate data collection cost. In short, compared to fully-supervised [5] and weakly-supervised [6] algorithms, semi-supervised algorithms can use any kind of labeled image data, meaning that the cost of data collection is moderate, which has become a point of interest in the field of image segmentation.

The current semi-supervised instance-segmentation algorithms can be divided into two categories. One uses weakly-supervised learning to generate pseudo-segmentation labels and applies them for model training. The other uses a transfer-learning algorithm to achieve semi-supervised image segmentation. For the first type of algorithm, Li et al. [7] and Bellver et al. [8] used object-detection labels and a pre-trained instance-segmentation model to generate pseudo-instance-segmentation labels, and used these pseudo-labels to implement semi-supervised instances. However, the accuracy of segmentation prediction was still relatively large compared with the fully-supervised models. For the second type of algorithm, the $Mask^{X}R-CNN$ algorithm [9] uses transfer learning to achieve semi-supervised instance segmentation; it achieved a good semi-supervised instance-segmentation effect on the COCO dataset, but it cannot make full use of the source task characteristics to optimize the process of migration learning. Therefore, existing semi-supervised methods have two challenging problems feature losses and long training time.

In order to solve these problems, this work proposes a feature transfer semi-supervised instance-segmentation model based on category attention to improve the performance of instance segmentation. To address the first problem, we retain input data features by designing a migration-optimization module based on a category attention mechanism. For the second problem, we develop a two-branches structure, which has an object-detection branch and a segmentation-prediction branch. We use a migration-optimization module to connect the two branches for training. Meanwhile, to incorporate attention features into the AFT-Mask model, we adopt a consistent activation function between the migration-optimization module and the segmentation prediction.

The contributions of this work are as follows:

1. In order to solve the problem of feature losses, this article proposes a category attention-based feature-migration-optimization module. It avoids the sparsity of one-hot vectors and retains the information of the feature map by using the result of RoI feature classification as the category attention. Furthermore, to solve the problem of inconsistent activation functions after introducing the category-attention module, we standardize the category-attention module by adopting the sigmoid activation function to enhance segmentation-prediction accuracy.
2. Aiming to decrease the training time, we connect the feature-migration and segmentation-prediction modules by using an inverse-convolutional module. The benefit is that the training process of the object-detection branch can directly benefit from the backpropagation of the segmentation-prediction error.

This article is composed of four parts. The second chapter introduces work related to this article. The third chapter—Materials and Methods—describes the overall structure of the AFT-Mask model, the specific implementation details of the category-attention module and its advantages over the TransferNet attention module. The fourth chapter compares the effects of different network structures on the performance of the AFT-Mask model. It also analyzes the difference in the performance of semi-supervised instance segmentation between some of the best semi-supervised or fully-supervised instance-segmentation models. The last part is the conclusion of this work.

## 2. Related Work

There are two kinds of methods for implementing semi-supervised algorithms in the field of image segmentation. One method is to use weak supervised learning to generate pseudo-segmentation labels for model training. The other method is to use the migration-learning algorithm to achieve semi-supervised image segmentation. For example, Li [7] and Wei [10] used an image dataset with “weak labels” to generate pseudo-segmentation labels for semi-supervised instance segmentation. Hu [9] and Li [11] used migration learning to complete semi-supervised instance segmentation. The pseudo-semantic segmentation mask generated by the weak supervised semantic-segmentation method can be better used for semi-supervised learning. However, weak supervised instance-segmentation algorithms have been widely studied in recent years, but the quality of the pseudo-instance-segmentation mask generated by current models is still poor. Therefore, the performance of semi-supervised instance-segmentation algorithms based on pseudo-segmentation mask generation is still not good enough [8]. In the study of semi-supervised instance segmentation, Bellever [8] and others used traditional image segmentation algorithms and pre-trained models to generate pseudo-instance-segmentation labels. Due to the limited quality of generated pseudo-segmentation labels, the accuracy of semi-supervised instance segmentation is very low. With the progress of research on weak supervised instance-segmentation algorithms, it is expected that there will be more semi-supervised instance-segmentation algorithms based on pseudo-instance-segmentation labels.

Another direction of interest is the integrating of the migration learning module into semi-supervised instance segmentation algorithms. Hu R et al. [9] used the parameter migration method in migration learning to design a semi-supervised instance segmentation algorithm, and achieved a good instance segmentation effect on the COCO dataset. The tasks in the field of computer vision are diverse and interrelated among many tasks [12]. The migration learning method can use the knowledge learned from different tasks to complete the target task. However, the feature mapping information of the migration module is still lost, so there is a lot of room for the development of the semi-supervised instance segmentation algorithm based on migration learning.

### 2.1. Migration Learning

In machine learning, migration learning refers to the use of algorithms to accomplish a task on the “source domain” to improve its effectiveness in completing the target task on the target “data domain”. Strictly speaking, “data domain” refers to the input data feature space X and its edge probability distribution P (X), and “task” refers to the task label variable space Y and the corresponding conditional probability distribution P (Y|X). Therefore, the migration learning algorithm is applied to situations where the source data domain is different from the target data domain, or where the source task is different from the target task. Different “domains” include different characteristic spaces for input data (e.g., Chinese material data in document processing tasks and English language data) and different edge probability distributions for input data (e.g., natural scene image and indoor scene image datasets). Different “tasks” include different feature spaces for task labels (e.g., image classification and object detection). The conditional probability distribution of task labels varies (e.g., single-category label classification issues and multi-label classification issues).

Migration learning has been studied in the field of machine learning for more than 10 years. According to the summary of Pan S J et al. [13] and Tan C et al. [14], the migration learning approach consists of four main aspects. (1) Instance knowledge migration: reset the weight of supervised data in the source data domain and use part of the source domain’s data for the target data domain. This method can take advantage of the correlation between the source data domain and the target data domain to enhance the model’s learning in the target task. (2) Feature expression migration: discover a “good” feature expression to reduce the difference between the source domain and the target domain and the error of the algorithm model. Feature expression is the embodiment of the knowledge that the model learns in the source domain and corresponding source tasks, so these feature expressions can help the algorithm to accomplish the target task better to some extent. (3) Parameter migration: the migration learning benefits from the use of shared parameters or a priori in the source and target domain models. (4) Relationship knowledge migration: build a mapping of source and target domain relationship knowledge. Similar to instance knowledge migration, this approach takes advantage of the correlation between source and target domain data.

Although the migration learning process can be implemented by different methods, the above migration learning methods have their specific ideological principles and application conditions, and the correct use of these methods can help the algorithm model to better accomplish the target task. By studying the migration learning algorithm and the related semi-supervised instance segmentation algorithm, this work adopts the migration learning method based on feature expression migration to achieve semi-supervised instance segmentation and puts forward a novel feature migration optimization algorithm.

### 2.2. Attention Mechanism

In deep learning, attention mechanisms are often used in the field of natural language processing from sequence to sequence model optimization [15,16]. Attention mechanism also appears from time to time in the field of image research [17,18,19,20,21]. Among them, the TransferNet algorithm [17] uses category attention to migrate learning semantic segmentation models, so that models learn specific segment masks for different category characteristics. The feature migration optimization module in this work draws on the design of the TransferNet attention module, but they differ greatly in network structure and usage methods. The literature list of instance segmentation algorithms is shown in Table 1.

## 3. Materials and Methods

This work studies the task of using attention mechanism and migration learning ideas to achieve semi-supervised instance segmentation and designs AFT-Mask, a semi-supervised instance segmentation model based on feature migration. This section begins with an introduction to the overall structure of the AFT-Mask model, then describes the design and implementation details of the AFT-Mask model category attention module.

### 3.1. The Overall Structure of the AFT-Mask Model

**Figure 1:**
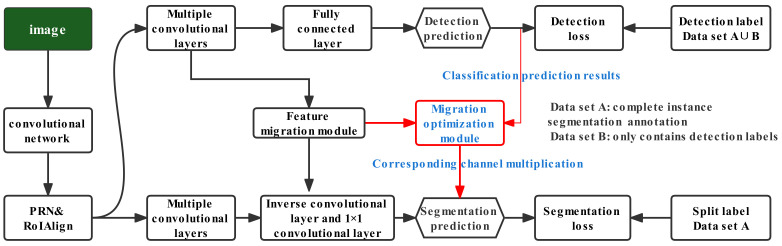
The overall structure of the AFT-Mask model.

Figure 1 shows the network structure of the AFT-Mask model presented in this work, which uses the basic network as The Mask R-CNN, the existing full-supervised model with better performance in the field of instance segmentation. The AFT-Mask model extracts visual features from the image in the shared network section and then multitasks through the object detection branch and segmentation prediction branch, with the feature migration branch in the middle part of Figure 1. As shown in the figure, the migration optimization module in this work uses the classification prediction results of the object detection branch output to optimize the output characteristics of the feature migration module. When encoding implementations, the size of the classification prediction tensor is m×r×c, where m represents the number of batch images, r represents the number of areas of interest, and c represents the total number of categories classified. The output feature tensor size of the feature migration module is m×r×h×w×C, where *m* and *r* mean the same as above, and *h*, *w*, and *C* represent the height, width, and the number of channels of the feature map, respectively. The *r* area of interest for the classification prediction results and feature migration module output features corresponds to the index, so the category prediction value of the classification prediction results for each area of interest effectively provides category attention.

The output of the AFT-Mask model’s overall migration learning branch will be directly integrated with the prediction results of the segmentation prediction branch. The segmentation prediction branch of the model uses the sigmoid activation function to map the value of the segmentation mask pixel to an interval of 0 to 1. To ensure that the pixel value is still within that interval, the output of the migration branch is connected to the segmentation prediction result by multiplying the corresponding channel. Furthermore, to complete the corresponding channel fusion process described above, the output of the migration optimization module must be the same as the output size of the segmentation prediction module. The flowchart of our method is shown in Algorithm 1.
**Algorithm 1 **Algorithm flowchart.**Input: **images**Output: ** segmented instances  1:Convert input images into a digital matrix  2:Input the matrix into the AFT-Mask model as shown in Figure 1  3:Output segmented instances

### 3.2. Category Attention Module Design

**Figure 2:**
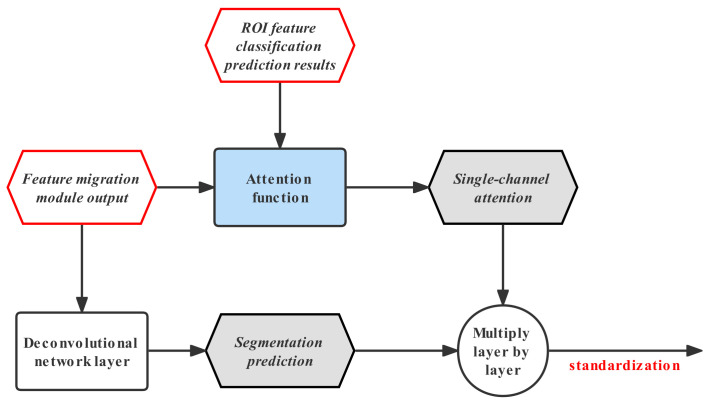
The structure of the migration optimization module based on category attention.

The attention mechanism is often used in natural language processing algorithms, and is also used in the field of image segmentation. For example, the TransferNet model designed by Hong S et al. uses category attention to implement weakly supervised semantic segmentation based on transfer learning. For the feature transfer optimization scenario in this article, the attention mechanism module of the TransferNet model has the following problems. Firstly, the category one-hot encoding is particularly sparse, and the dilution of the input features after its participation in feature conversion will be more serious. Secondly, its standardization process uses the soft-max function, which is inconsistent with the activation function used in the segmentation prediction of the AFT-Mask model, so it cannot be used in the feature migration optimization module of this article. The category attention feature migration optimization module proposed in this work solves the above-mentioned problems of the TransferNet attention module. The structure of the module is shown in Figure 2. As can be seen from Figure 2, the module is divided into the attention mechanism module and an inverse convolutional network module. The attention mechanism module uses target-related category information to provide “attention” for input features and converts the input feature map into a single-channel feature map. The inverse convolutional network module outputs a feature map with the channel size equal to the number of categories and provides an output mask with the same resolution as the segmentation prediction result for fusion with the segmentation prediction result. The input of the attention function is the output feature map of the feature transfer module and the prediction result of RoI feature classification.

Different from the TransferNet model, the model in this work uses the result of RoI feature classification as a simulation of category attention. The above approach has three benefits. Firstly, its value is a floating-point number in the range of [0, 1], which avoids the sparsity of the one-hot vector, and can better retain and convert the information of the migration module feature map than the one-hot value. Secondly, the output feature comes from the conversion of the same RoI feature, so the feature map for each target area has inherent independence. Thirdly, the feature classification result is the prediction of the target classification branch for the target category of the region to which the feature map belongs, so it can be used as a substitute for the category label.

(1) $$att^{r}=RoIupsampling\left(\sum \kappa w_{k}^{r}*conv2d{\left(feat^{r}\right)}_{k}\right)\;$$

Formula (1) represents the calculation process of the attention function proposed in this work. The input feature map of each region of interest in the formula is transformed into a category feature map $feat^{r}$ by a convolution function. The resolution of the category feature map is consistent with the input feature map, and the number of channels is the same as the number of classification task categories. For the features of multiple different channels, the accumulation function uses the corresponding category prediction values to sum their weights, then a single-channel feature map is obtained. To make the output feature resolution the same as the inverse convolution output feature, the upsampling function enlarges the single-channel feature size to twice the original size.

The feature conversion part before the upsampling function in Formula (1) is similar to the generation formula (see Formula (2)) of category activation map [24] commonly used in weakly supervised image segmentation models, but they have essential differences. The category activation map uses the weight of the network layer to weight the corresponding output feature map of the convolutional layer, but the feature map of the attention function in this article is not directly related to the category prediction value, and the performance of the attention function in this article needs to be in the supervised training process in the promotion.

(2) $$M_{c}\left(x,y\right)=\sum \kappa w_{k}^{c}*f_{k}\left(x,y\right)\;$$

The feature migration optimization module of this work needs to use input features for semantic segmentation mask prediction. The TransferNet model uses the soft-max activation function as a feature standardization method, but it is inconsistent with the sigmoid activation function used in the original segmentation prediction branch. In order to ensure the effectiveness of segmentation prediction, the standardization of the model migration optimization module in this work uses a sigmoid activation layer. The feature maps of the two branches are multiplied by the corresponding elements of the feature maps layer by layer in the merged part of the network module. The layer-by-layer multiplication operation is used here instead of the addition operation because the multiplication operation can provide richer information for the attention feature map [17].

### 3.3. Model Implementation Details

The attention function of the migration optimization module first needs to use a convolution operation to transform the output characteristics of the migration module. The size of the convolution kernel used in the convolution operation of the attention function is 1 × 1, the padding is 0, the step size is 1, and the number of output channels is the same as the number of categories of the classification branch. The reason for using a convolution kernel with a size of 1 × 1 instead of 3 × 3 is that there is no need to extract the visual information contained in the convolution feature again, only channel aggregation of the features. Additionally, the 1 × 1 size convolutional layer has relatively fewer parameters, which is easier for network training. The upsampling process in the attention function uses bilinear upsampling, and the size of the feature map after sampling is twice the original. Since the overall structure of the feature transfer branch is relatively complex, the use of bilinear upsampling in the attention function can reduce the training parameters of the feature transfer branch, making the overall feature transfer branch easier to optimize. The size of the convolution kernel used by the inverse convolutional network layer of the migration optimization module is 2 × 2, the padding is 0, the step size is 2, and the number of channels is equal to the number of categories. The layer-by-layer multiplication process in the feature optimization module needs to multiply the position-aligned feature value of the single-channel feature map output by the attention mechanism branch and the feature map of each channel output by the inverse convolutional network layer.

In the coding implementation process of the AFT-Mask model, the inference stage of the model needs to solve the problem caused by the difference in the number of output features of the Mask R-CNN basic model object detection branch and the number of input features of the segmentation prediction branch. The Mask R-CNN model in its inference stage first filters the RoI output by the RPN network through the NMS algorithm according to the results of the object detection and then uses the corresponding RoI feature as the feature input of the segmentation prediction branch (the number of features does not exceed 100 by default), and its object detection branch input feature is the normal number (default 1000). The feature transfer branch of the AFT-Mask model takes the object detection feature as input, and the number of output features is also consistent with the object detection branch. Therefore, the output feature of transfer branch and the feature of segmentation prediction branch have a problem that the number does not match.

The AFT-Mask model customizes the feature filter layer in the inference stage to filter the output features of the migration branch. The feature filtering layer uses the same strategy as the RoI filtering process of Mask R-CNN, and filters the output features under the same index. The migration features retained by the feature filter layer are consistent with the number and index of the input features of the segmentation prediction branch, so adding the feature filter layer solves the above problem.

## 4. Experimental Results

This section first describes the semi-supervised dataset division, model training methods and basic experimental settings. Then, compare the impact of different network structures on the performance of the AFT-Mask model. Finally, compare the semi-supervised instance segmentation performance difference between AFT-Mask and some current best semi-supervised or fully-supervised instance segmentation models.

### 4.1. Semi-Supervised Dataset Division

The AFT-Mask model is semi-supervised training on the well-known public instance segmentation dataset COCO2017 in the field. The training set of this dataset has 118,287 images, which requires a high cost of time. The experiment cost can be saved by reducing the amount of training data, but a small sample size will lead to unbalanced training data categories. Therefore, in this section of the experiment, 25% of the COCO training set data with complete instance segmentation labels are randomly selected as sub-training set A, and 25% of the COCO training set data with object detection labels are added as sub-training set B. The COCO instance segmentation verification set is used as the test dataset for this experiment. To sum up, in the dataset division method of this article, the training data with complete instance segmentation labels accounted for 25% of the total data volume of the COCO training set, and the training data with object detection labels (A⋃B) accounted for the total COCO training set 50% of the data volume, so the semi-supervised dataset division method in this article is feasible. For ease of explanation, the dataset divided in this article will be referred to as the COCO-25% semi-supervised dataset in the following text.

The $Mask^{X}R-CNN$ algorithm related to this article uses the “20/60” dataset division rule, where “20” represents the COCO sub-dataset (that is, voc subset) that contains 20 object categories in the Pascal VOC dataset. The “60” represents the COCO sub-dataset (i.e., non-voc subset) containing the remaining 60 object categories. According to the work $Mask^{X}R-CNN$, the training set and test set of COCO2017 need to be divided into different A and B sub-datasets according to the “20/60” division rule. Specifically, there can be two division methods. On one hand, A represents “voc subset”, B stands for “non-voc subset”. On the other hand, A stands for “non-voc subset” and B stands for “voc subset”.

The A and B sub-datasets in this article contain the same label types as $Mask^{X}R-CNN$, but the instance segmentation annotations of the images in the sub-dataset A in this article cover 80 COCO object categories, and the sub-data in $Mask^{X}R-CNN$ as described above A only contains some COCO categories. Therefore, the COCO-25% semi-supervised dataset division method will not cause the “category difference” problem for the model training process [9], and there is no need to design the training process similar to the “stop grad” in the $Mask^{X}R-CNN$ training method. In addition, the amount of semi-supervised datasets in this work is relatively smaller, and the experimental time cost of the model is relatively lower.

The training method of the AFT-Mask model is slightly modified on the training method of $Mask^{X}R-CNN$. The method is specifically divided into two processes: the first process is phased training, and the second process is joint training. Among them, the phased training process is the same as the phased training process of the $Mask^{X}R-CNN$ algorithm, while the joint training process is different from the “end-to-end joint training” process of the algorithm.

This method first divides the COCO2017 dataset into two sub-datasets A and B for semi-supervised training. The images in sub-dataset A have complete instance segmentation annotations, and the images in sub-dataset B have only category and border annotations. The training of the model in this work adopts the A and B sub-dataset division of the COCO-25% semi-supervised dataset division method in the previous section.

### 4.2. Experimental Settings

In the experiment, each model is trained through a high-performance GPU (Graphic Processing Unit) server, which is configured by an NVDIA 1080Ti graphics card with 12 G video memory. The Mask R-CNN research and $Mask^{X}R-CNN$ research related to the research in this article used 1024×1024 images as input during training and used 8 GPUs with 12 GB memory capacity to train the model. Each GPU simultaneously trains two images, but this training method requires a higher cost. Based on the research time cost limitation, the input image size of this experiment is 512 × 512, and the GPU trains 4 images at the same time each time. Because the resolution of the input image is relatively small, the experiment reduced the size of the feature map output by the RPN network to the same proportion, and set the threshold of the NMS algorithm of the RPN network to 0.6. The Mask R-CNN model and $Mask^{X}R-CNN$ use the model’s initial learning rate (learning rate) to be set to 0.02, and their models are implemented using the Caffe framework. However, the bottom layer of the model code in this article is based on the TensorFlow framework. According to related research [25], it is pointed out that the optimization function of TensorFlow is different from that of Caffe. The learning rate of 0.02 will lead to the problem of gradient explosion in model training. Therefore, according to the recommendation of the research, this work sets the initial learning rate of the model to 0.001, and the initial learning rate of the joint training process to 0.0001. For the convenience of description, the hyperparameter setting proposed here is called the “crop” strategy in this article.

### 4.3. The Influence of Network Structure

The feature migration optimization module based on category attention proposed in this work is composed of an attention function part and an inverse convolutional network part. Therefore, this section explores the influence of network structure on model performance through experiments. In the first training phase of the phased training process, the weight of the segmentation prediction loss is set to 0.

**Figure 3:**
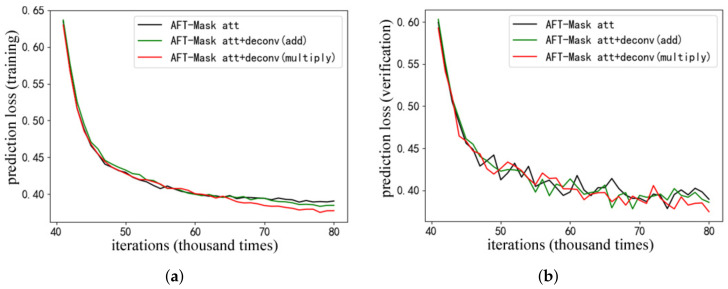
Variation of segmentation prediction loss with the number of iterations. (**a**) Comparison of training losses. (**b**) Comparison of verification losses.

Figure 3 shows the variation of the segmentation prediction loss with the number of iterations in the experiment. Figure 3a shows the variation of the segmentation prediction training loss, and Figure 3b shows the variation of the segmentation prediction verification loss. The 41 K to 60 K iterations and the 61 K to 80 K iterations are the second stage of the phased training process and the joint training process, respectively. Finally, the model segmentation prediction training loss has remained flat, which shows that the model is approaching convergence. Due to the limitation of experimental cost, the segmentation prediction verification loss in the training process only tests 50 steps of image data (about 200), so the segmentation prediction verification loss in Figure 3b shows a certain range of fluctuations.

In Figure 3, the AFT-Mask model with only the attention function part is named AFT-Mask att, and the AFT-Mask model with the attention function part and the inverse convolutional network part at the same time is named AFT-Mask att+deconv. According to the combination of addition and multiplication of the feature migration optimization module mentioned above, the AFT-Mask att+deconv model is further divided into the AFT-Mask att+deconv(add) model and the AFT-Mask att+deconv(multiply) model. It can be seen from the figure that the segmentation prediction training loss of the AFT-Mask att+deconv (multiply) model is significantly lower than other models after about the 65 K iteration. After about 70 K iterations, the training loss of the AFT-Mask att+deconv(add) model is significantly lower than that of the AFT-Mask att model. Its overall trend is consistent with Figure 3a, although the segmentation prediction verification loss fluctuates in Figure 3b. It can be inferred from the change of segmentation prediction loss that the feature migration module proposed in this work is more helpful to improve the performance of the AFT-Mask model when it has a complete network structure and uses channel multiplication to connect substructures.

Table 2 shows the results of the instance segmentation test on the COCO verification set of the AFT-Mask models with different structures. The performance evaluation indicators are the mAP and $AP_{50}$ indicators commonly used in the field of instance segmentation. It can be seen from the table that the AFT-Mask att+deconv (multiply) model is higher than other models in the two main performance indicators of mAP and $AP_{50}$. Therefore, combining the segmentation prediction loss changes in Figure 3 and the accuracy test results in Table 2, it can be inferred that the AFT-Mask model with a complete structure and connection using channel multiplication has a stronger semi-supervised instance segmentation capabilities.

### 4.4. Comparisons with the Divided Semi-Supervised Dataset

**Figure 4:**
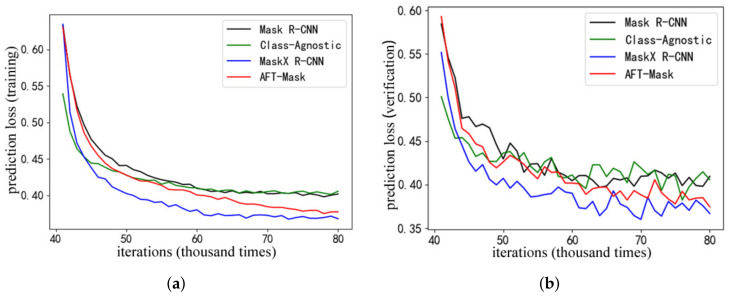
Comparison of prediction loss with iteration number by correlation model segmentation. (**a**) Training loss. (**b**) Validation loss.

To verify the effectiveness of the transfer learning of the AFT-Mask model, this section compares it with the Mask R-CNN benchmark model for semi-supervised instance segmentation performance. In addition, this section also compares the performance of other related models in the AFT-Mask model field. The above models are all trained using the COCO-25% semi-supervised dataset division method and training method in this article. This section reproduces the unknowable Mask R-CNN and $Mask^{X}R-CNN$ models based on the open-source Mask R-CNN model code and the implementation instructions of related models. Figure 4 shows the comparison of changes in the segmentation prediction loss of each model with the number of iterations during the training process. The class-agnostic model in Figure 4 is the class-agnostic Mask R-CNN model. This model is used as the benchmark model in the research of $Mask^{X}R-CNN$, so this article will use it as a comparison object. Other models include Mask R-CNN, $Mask^{X}R-CNN$ and AFT-Mask in this article. The first 40 K iterations of the training process are object detection branch training, and the weight of the segmentation loss is set to 0. The segmentation prediction loss in this process is not recorded, so the figure only shows the segmentation prediction loss of the last 40 K training. It can be seen from the training segmentation prediction loss in Figure 4a that the training loss of the AFT-Mask model is significantly lower than the training loss of the class-agnostic model and Mask R-CNN model from 60 K to 80 K iterations. Higher than the training loss of $Mask^{X}R-CNN$ model. Since the verification set image used in the training process is a small part of the overall verification set, the verification loss shown in Figure 4b fluctuates, but it can be seen from the figure that the segmentation prediction verification loss change is roughly the same as the training loss The trend of change.

After the above model is semi-supervised training under the COCO-25% semi-supervised dataset division method and training method proposed in Chapter 3 of this article, the instance% semi-supervised dataset divided in this work only contains 25% of the instance segmentation annotation data of the COCO training set, so the instance segmentation test accuracy in Table 3 is overall low.

It can be seen from the Table 3 that the AFT-Mask model is higher than the Mask R-CNN benchmark model, the class-agnostic model and the FT-Mask model in general instance segmentation accuracy indicators, such as mAP, $AP_{50}$, $AP_{75}$, and $AP_{S}$. From the experimental results, it can be seen that the AFT-Mask model effectively transforms the knowledge of the object’s visual features learned by the object detection branch through the feature transfer process, which helps improve the performance of model segmentation prediction. The feature transfer optimization module of the AFT-Mask model improves the learning performance of the feature transfer branch by providing category attention for the feature transfer branch. That is, combining migration learning can make our AFT-Mask model have better performance.

## 5. Conclusions

In the experiments of this work, the AFT-Mask model performs better than the Mask R-CNN benchmark model under the COCO-25% semi-supervised dataset and the proposed training method, so the feature transfer learning process is effective. In the A, B sub-dataset division method and training mode used by $Mask^{X}R-CNN$, the semi-supervised instance segmentation accuracy of the AFT-Mask model is higher than that of the Mask R-CNN class-agnostic benchmark model, but it is similar to the $Mask^{X}R-CNN$ model. There is still a certain gap in performance. The sub-dataset division method and training mode of $Mask^{X}R-CNN$ focus on the verification of model category generalization, so the AFT-Mask model has room for improvement in category generalization issues.

The model in this work can achieve effective semi-supervised instance segmentation, but there are still areas worthy of improvement. In future research work, there can be the following expansion directions. On one hand, future work can separately study the impact of category annotation data, target border annotation data, and supervised training instance segmentation annotation data on the model performance, and design a more general and more efficient semi-supervised instance segmentation model. On the other hand, researchers can consider designing category-independent feature migration and related optimization algorithms to improve the category generalization of the model.

## Figures and Tables

**Table 1:** Literature list of instance segmentation algorithms.

Name	Time	Method Type
Lin et al. [2]	2014	fully-supervised
Zhang et al. [3,4]	2021	fully-supervised
Wang et al. [5]	2022	fully-supervised
Li et al. [7]	2018	weakly-supervised
Bellver et al. [8]	2019	weakly-supervised
Liu et al. [6]	2021	weakly-supervised
Papandreou et al. [22]	2015	semi-supervised
Wei et al. [10]	2018	semi-supervised
Lee et al. [23]	2019	semi-supervised
Hu et al. [9]	2018	migration learning + semi-supervised
Zamir et al. [12]	2018	migration learning + semi-supervised
Li et al. [11]	2019	migration learning + semi-supervised
**AFT-Mask**	2022	**attention + migration learning + semi-supervised**

**Table 2:** Comparison of segmentation accuracy of AFT-Mask model examples with different structures.

Model Name/Precision	mAP	${\mathrm{AP}}_{\left(50\right)}$
AFT-Mask att	10.8%	27.3%
AFT-Mask att+deconv(add)	10.9%	27.6%
AFT-Mask att+deconv(multiply)	11.1%	28.0%

**Table 3:** Comparison of model performance under partitioning of semi-supervised dataset and training method in this paper.

Model /Indicators	mAP	${\mathrm{AP}}_{\left(50\right)}$	${\mathrm{AP}}_{\left(75\right)}$	${\mathrm{AP}}_{\left(S\right)}$	${\mathrm{AP}}_{\left(M\right)}$	${\mathrm{AP}}_{\left(L\right)}$
Mask R-CNN	10.2%	25.7%	6.4%	3.5%	10.9%	15.9%
Class-Agnostic	10.1%	25.5%	6.3%	3.4%	10.7%	15.7%
$Mask^{X}R-CNN$	11.6%	29.3%	7.0%	3.7%	12.1%	19.2%
AFT-Mask	11.1%	28.0%	6.7%	3.6%	11.6%	18.0%

## Data Availability

Data available in a publicly accessible repository that does not issue DOIs Publicly available datasets were analyzed in this study. These data can be found here: [https://pan.baidu.com/s/1hNuGuR43Gi3A59w5Jgd9_w?pwd=r89t], accessed on 9 November 2022.
